# Three Grand Challenges in High Throughput Omics Technologies

**DOI:** 10.3390/biom12091238

**Published:** 2022-09-04

**Authors:** Prashanth Suravajhala, Alexey Goltsov

**Affiliations:** 1 Amrita School of Biotechnology, Amrita Vishwavidyapeetham, Amritapuri, Clappana, Kerala 690525, India; 2 Bioclues.org, Hyderabad 500072, India; 3 Biocybernetics Systems and Technologies Division, Institute for Artificial intelligence, MIREA–Russian Technological University, 119454 Moscow, Russia

Over the years, next-generation sequencing (NGS) and advanced bioinformatics approaches have allowed the transition of genomic assays into translational practices. Emerging genomic assay technologies include characterizing mechanisms of disease prevalence, loss of heterozygosity tests, genotype-driven therapies, checking patient’s risk of disease recurrence, chromatin landscape, and gene expression signatures, to name a few [1]. On the other hand, there are vivid post-diagnostic risk assessment tools facilitating risk classification which would bring personalized medicine to the fore. Many professional societies and associations such as the American College of Medical Genetics and Genomics (ACMG) and the Association for Molecular Pathology (AMP) have developed a genomics curriculum framework to establish disease causality using functional assays [2]. With the emerging post-genomic assays in the realm of NGS, biochemical and functional assays can be better achieved through orthogonal checks for high throughput omics (HTO) approaches.

In this Special Issue, two articles have facilitated such vivid use of HTO approaches. These papers are also excellent examples of the systems genomics application, where bioinformatics is closely combined with in silico analysis of omics data, functions, networks, and pathway enrichment analyses aimed to investigate the molecular mechanisms of diseases and develop predictive and prognostic biomarkers. Srivastava et al. [3] performed whole genome sequencing in patients with Familial Non-Medullary Thyroid Cancer (FNMTC) to identify possible disease-causing germline variants in each family. The authors defined a small set of genes associated with FNMTC by prioritizing, ranking, and filtering the identified variants using the developed Familial Cancer Variant Prioritization Pipeline. It allowed them to further develop biomarkers of predisposition to FNMTC. This important result of the analysis may help in the identification of FNMTC-prone families which is a critical step in cancer risk assessment, cancer screening, and the development of cancer prevention strategy. In order to identify key biological functions and signaling pathways affected in FNMTC, authors carried out a pathway and network analyses and obtained that the GPCR, RTK, PI3K/AKT, and MAPK/ERK signaling pathways play a central role in the FNMTC. This finding may facilitate drug therapy targeting these pathways in FNMTC patients. Moreover, the authors established the similarity between deregulated pathways in FNMTC patients and these in other cancers that may promote drug repurposing. The important result of this work is also the established relationship between the proposed model for the molecular mechanisms underlying FNMTC and the reported mechanisms in non-familial NMTC. This analysis may help to elucidate the difference between more aggressive (FNMTC) and less (non-familial NMTC) aggressive cancer phenotypes based on genotype data.

The second paper [4] is devoted to the application of systems genomics to further elaborate the molecular mechanism of breast cancer based on in silico analysis of gene expression microarray of estradiol- and tamoxifen-treated samples. A wide arsenal of bioinformatics tools was used to perform the pathway and gene ontology enrichment analysis, construction of protein-protein interaction network, module analysis, construction of target genes-miRNA interaction network, and target genes-transcription factor interaction network. This systems approach and comprehensive analysis of drug action on the different levels of cell organization may promote the further elaboration of the signaling model of drug treatment mechanism and the development of new predictive biomarkers for biomarker-guided targeted therapies.

We discuss three grand challenges to check this: (a) Equal access to genomic testing by bridging the gap between clinicians and geneticists (b) Cataloging variants of unknown/uncertain significance and (c) Theranostics.

It is generally accepted that the NGS approaches have heralded the HTOs enabling studies from whole-genome, whole-exome sequencing approaches to single cell and spatial biology, among others [5,6] This has introduced the possibility of assembling a multitude of genomic tests in a cost-effective manner. There are approaches applied for detecting a number of somatic/germline genome alterations, those including structural variants, and chromosomal rearrangements. With levels of comparison of single nucleotide polymorphisms (SNPs) and single nucleotide variants (SNVs), the compendium of variants found in diseased patients is under evaluation. Such patients need genome testing and so is the gap to be bridged between a geneticist and a clinician during counseling. Current mutations in rare diseases could steadfastly be classified from variants of unknown significance (VOUS)/likely pathogenic to driver or passenger mutations as in cancer mutations. Although experimental models need to be established for this, a standard guideline for point-of-care testing would be a highly practical overview of the discovery process [7].

The VOUS has been of immense interest ever since the capture technology spanned beyond the vast majority of inferring disease-causing mutations. As mutations identified in non-coding regions may in fact be candidate drivers, interpreting the mutations at exon-intron boundaries with large numbers of VOUS has gained significance [8]. This has allowed researchers to generate new sequence alignments, and methods to interpret the functions. One needs a cursory examination to look into potential VOUS, and yet turn out to be significant and pathogenic. This allows us to ask pertinent questions about whether the sequencing is deep and whether the capture technologies are inherent to pathogenic mosaicism. At this point, non-synonymous mutations effectively are to be included among those swaths of genomic variant pool albeit the fact that they need to be thoroughly validated. Conversely, visual analysis and validation of reading alignment would allow us to identify potentially significant mutations to mechanistic pathways which is a reasonable challenge [9].

The greatest challenge, however, is to take up these avenues to the translational phase, a.k.a theranostics. It is worth considering assessing the significance and drawing conclusions from simulated variants of several patients from existing datasets. Invariably, what is, in theory, need not fit practically but what has been practically validated could be taken to the next futuristic course in the development of panels. Thanks to spatial genomics, nanostring, and a plethora of genomics tools that are available [10,11]. Nevertheless, a careful assignment of statistical threshold should be taken into account for suggestive functional significance in lieu of the development of NGS panels. While the sample size is a challenge, the idea of taking up the VOUS and gene testing panels would allow us to have an accurate development of the theranostic approach. 

HTO technologies served as a never-ending journey to discover the ideal needs of systems genomics wherein studying the variation alone of every individual forms an umbrella challenge. Added to this, with continuous improvement of individual variation, the need for bridging the gap between clinicians and geneticists, and further taking the baton to theranostics is the need of the hour. We firmly believe that future research will rely on these three inherent challenges but will possibly expand the outcomes for a holistic view in reaching therapeutics, diagnostics, and a better understanding of systems genomics. The ongoing efforts in developing new tools would possibly enable the consolidation of variants to capture the evolutionary state of the mutations. This can only be applicable if cross-disciplinarians in HTOs join hands for the development of consortia efforts. Soon, this will perhaps fill the gaps as we set a mark to catalog new challenges during the next decade. Can we? Together we Can.
